# Different Drug Mobilities in Hydrophobic Cavities of Host–Guest Complexes between β-Cyclodextrin and 5-Fluorouracil at Different Stoichiometries: A Molecular Dynamics Study in Water

**DOI:** 10.3390/ijms25115888

**Published:** 2024-05-28

**Authors:** Giuseppina Raffaini, Stefano Elli, Michelina Catauro, Antonio D’Angelo

**Affiliations:** 1 Department of Chemistry, Materials, and Chemical Engineering “Giulio Natta”, Politecnico di Milano, Piazza L. Da Vinci 32, 20131 Milano, Italy; 2 INSTM, National Consortium of Materials Science and Technology, Local Unit Politecnico di Milano, 50121 Milano, Italy; 3 Istituto di Ricerche Chimiche e Biochimiche ‘G. Ronzoni’, Via Giuseppe Colombo 81, 20133 Milano, Italy; elli@ronzoni.it; 4 Department of Engineering, University of Campania “Luigi Vanvitelli”, Via Roma 29, 81031 Aversa, Italy; michelina.catauro@unicampania.it (M.C.); antonio.dangelo@unicampania.it (A.D.)

**Keywords:** 5-fluorouracil, cyclodextrin, inclusion complexes, anticancer drug, molecular dynamics, solubilization, release mechanism

## Abstract

Cyclodextrins (CDs) are cyclic oligosaccharides able to form noncovalent water-soluble complexes useful in many different applications for the solubilization, delivery, and greater bioavailability of hydrophobic drugs. The complexation of 5-fluorouracil (5-FU) with natural or synthetic cyclodextrins permits the solubilization of this poorly soluble anticancer drug. In this theoretical work, the complexes between β-CD and 5-FU are investigated using molecular mechanics (MM) and molecular dynamics (MD) simulations in water. The inclusion complexes are formed thanks to the favorable intermolecular interactions between β-CD and 5-FU. Both 1:1 and 1:2 β-CD/5-FU stoichiometries are investigated, providing insight into their interaction geometries and stability over time in water. In the 1:2 β-CD/5-FU complexes, the intermolecular interactions affect the drug’s mobility, suggesting a two-step release mechanism: a fast release for the more exposed and hydrated drug molecule, with greater freedom of movement near the β-CD rims, and a slow one for the less-hydrated and well-encapsulated and confined drug. MD simulations study the intermolecular interactions between drugs and specific carriers at the atomistic level, suggesting a possible release mechanism and highlighting the role of the impact of the drug concentration on the kinetics process in water. A comparison with experimental data in the literature provides further insights.

## 1. Introduction

Cyclodextrins (CDs) were discovered in 1891 by Villiers [1], and γ-CD was discovered in 1935. Although they have been known for 130 years, their first applications in the pharmaceutical and food industries only occurred in the 1980s [2,3]. They are considered to be molecular containers [4,5,6]. Cyclodextrins and materials containing cyclodextrins are likely to find increasing usage in a growing diversity of pharmaceutical applications, most notably those involving macromolecular therapeutics, as an interesting family of cage molecules [7,8,9]. Cyclodextrins, as drug delivery systems, have been extensively studied according to differences in the drug therapeutic environment, such as the pH, light, temperature, redox, and enzymes [10,11,12,13,14,15,16,17,18]. Native cyclodextrins and chemically functionalized CDs are smart tools that have been proven to be effective for use in the preparation of drug delivery systems, becoming a new area of research in recent years due to their ability to form stable host–guest inclusion complexes with poorly soluble drugs by encapsulating them in their hydrophobic cavities or interacting with them at the outer surface [19,20,21,22].

CDs are synthetic substances obtained from the enzymatic degradation of one of the most essential polysaccharides, starch, formed by α-1,4-linked glucose units. α-, β-, and γ-CDs are the most common CDs and include 6, 7, and 8 glucose units, respectively. α-CD and β-CD are the most common natural cyclodextrins. They have a truncated-cone shape with a hydrophilic outer surface and a hydrophobic inner cavity. The inner diameters of α-, β-, and γ-CDs are 4.7–5.3, 6.0–6.5, and 7.5–8.3 Å, respectively [1]. Cyclodextrins can sometimes form water-soluble inclusion complexes with small molecules or portions of large compounds in different stoichiometries depending on the size and concentration of the drug [23,24,25]. Recently, it was observed that CDs and CD complexes self-assemble to form nanoparticles [15,16] and that, under certain conditions, these nanoparticles can self-assemble to form microparticles. These properties have changed the way we perform CD pharmaceutical research and have led to new CD formulation opportunities, as recently summarized by Kurkov and Loftsson [24]. In general, negligible amounts of aggregates are formed in pure CD solutions, but the formation of aggregates is greatly enhanced with the formation of an inclusion complex, and the extent of the aggregation increases with an increasing concentration of CD [25]. These biocompatible cyclic oligosaccharides do not elicit immune responses and have low toxicities in animals and humans [26,27,28,29]. In native and modified CDs, the absorption process involves the uptake of the guest into the cavities of the CDs to form a host–guest inclusion complex, and it is different from adsorption whereby molecules attach to the surfaces of CDs. Complexation occurs in the CDs’ cavities or on their outer hydrophilic surfaces, where a large number of OH groups are present and can influence the kinetics of release [30,31].

CD-based nanomaterials for drug delivery include cyclodextrin nanosponges [32,33,34,35,36,37,38,39], cyclodextrin-based host–guest supramolecular hydrogels [40,41,42], porous networks for drug delivery systems [43,44,45], and cyclodextrin metal–organic frameworks [46]. The possibility of decreasing the complexation’s efficiency and solubilization effect with an increase in the amount of cross-linker, especially in cyclodextrin-based nanosponges, has been studied, especially when the amount of drug to be released must be small to avoid cytotoxic effects [21], particularly in anticancer therapy [47,48,49,50,51,52,53,54].

The drug studied in this theoretical work, 5-fluorouracil (5-fluoro-1H,3H-pyrimidine-2,4-dione (5-FU)), is an anticancer drug administered intravenously against colorectal or pancreatic cancer or as a cream for skin cancer [55,56,57]. 5-FU is poorly soluble in water (ca. 1 mg/mL) [58]. Appropriate carriers or substrates can enhance its bioavailability and mitigate the development of serious side effects [59,60,61,62,63,64,65,66,67,68,69,70,71]. Molecular simulations of the adsorption process of 5-FU on graphene oxide nanosheets as a drug delivery vehicle [72], MD simulations related to hydrophilic pores in phospholipid bilayers and Fe_3_O_4_ nanoparticles loaded with 5-FU [73], and investigations into the adsorption of 5-FU on gold clusters [74,75] have attracted attention. The theoretical approaches in materials science able to interface soft matter and molecules for the development of drugs for, as an example, biomedical applications, can help with the explanation of experimental data [44].

In the literature, in terms of CD and 5-FU inclusion complexes for drug delivery, theoretical studies based on docking, density functional theory (DFT) calculations, and molecular dynamics simulations at the atomistic level have indicated that stable inclusion complexes in a 1:1 stoichiometry can be formed [71,72,73,74,75,76,77,78,79,80,81]. Di Donato et al. [71] show how the complexations of 5-FU with α-CD or β-CD are able to determine a significant increase in the anticancer activity of this widely used drug. The 5-FU is a small molecule compared to the size of the β-cyclodextrin. It is important to highlight that a 1:2 stoichiometry is rare for β-CD. β-CD exhibits a relatively small cavity compared to the larger γ-CD, as reported in the literature by Clarke et al. [82,83]. Using DFT study with dispersion correction, Buczek et al. studied the interaction of 5-fluorouracil with β-cyclodextrin for understanding the interaction of 5-FU with β-CD in the solid state and also suggested the presence of equilibrium between β-CD/5-FU complex and solvated 5-FU [77]. Melnikova et al., using PFG NMR techniques, found strong evidence of the formation of the host–guest complex between β-CD and 5-FU in aqueous solution, which is likely to be an inclusion complex [84]. However, this complex is not stable, having a short lifetime. The authors explained that routine 1D and 2D NMR experiments are not sensitive enough for the detection and characterization of the β-CD/5-FU complex, probably due to the short lifetime of the species, as well as the mobility of 5-FU in the interior of the complex.

Based on the important information reported in the literature and experimental results, the aim of the present work was to theoretically study the possible host–guest complexes between β-CD and 5-FU in water both in 1:1 and 1:2 β-CD/5-FU stoichiometries. Using MM and MD simulations, we investigated the stability over time and mobility of 5-FU drugs encapsulated or far from the β-CD cavity. Due to the small size of the 5-FU, it is possible to include two 5-FU drugs in the hydrophobic β-CD cavity. The weaker van der Waals interactions and possible H-bonds among β-CD and 5-FU drugs in water must be further investigated to better understand the stability of complexes over time in different stoichiometries and the related diffusion properties of the encapsulated or “free” drug, away from the hydrophobic cavity of β-CD, in comparison with interesting recent NMR experiments reported in the literature [84].

## 2. Results and Discussion

**Figure 1:**
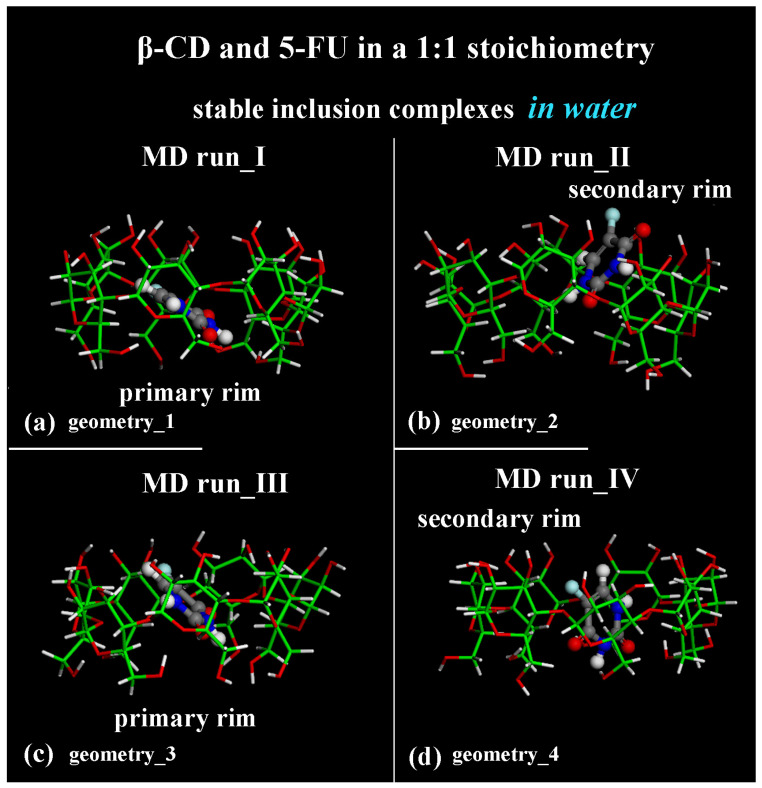
Side view of the optimized geometries after four MD runs, lasting for 2 ns, in water, with the 5-FU initially located near the primary rim or the secondary rim (see Figure S1a,c and Figure S1b,d, respectively). Cyclodextrin atoms are represented by sticks; 5-FU atoms are represented by balls and sticks. Color code: β-CD carbon atoms are indicated in green; carbon atoms of 5-FU in gray; oxygen in red; nitrogen in blue; fluorine in light blue; and hydrogen in white. Water molecules are omitted for clarity.

In this section, the theoretical study of the interactions between β-CD and the 5-FU drug molecule in 1:1 (see Section 2.1) and 1:2 (see Section 2.2) stoichiometries using MM and MD simulations at the atomistic level in water is presented and discussed. The optimized geometry of the β-CD used in the present work is the same as that studied by Raffaini et al. [78,79]. 5-FU is subject to tautomeric equilibria between several structural isomers [85,86], and only one of them (diketo C=O/C=O form) is present practically exclusively in aqueous solutions [87], and is also active as a chemotherapeutic agent [88,89]. This structure, reported in Figure 1 in Reference [84], is studied in this work. An additional study considering an initial geometry with the β-CD and two 5-FU drug molecules in a random arrangement lasting 10 ns is performed and finally discussed (see Section 2.3).

### 2.1. β-CD/5-FU Inclusion Complexes in a 1:1 Host–Guest Stoichiometry in Water

First, the intermolecular interactions between the β-CD molecule and the 5-FU drug molecule in a 1:1 stoichiometry in water, without the assumption of any a priori inclusion complexes, were studied. Using a simulation protocol adopted in previous works [30,78,79,90] described in Section 3, Materials and Methods, four different initial geometries, in which 5-FU was located far from the β-CD cavity and near the primary or secondary β-CD rim, were investigated in water. The four nonoptimized initial geometries, as reported in Figure S1, were considered.

After initial geometry optimizations, during MD runs lasting for 2 ns at 300 K the inclusion process occurred. In the host–guest inclusion complexes optimized at the end of MD runs when an equilibrium state was achieved and in numerous conformations assumed by the system during MD runs, the 5-FU drug molecules were encapsulated in the hydrophobic β-CD cavity. Interestingly, through an analysis of all frames periodically saved during MD runs every 5 ps, it was observed that for part of the simulation, the drug is well encapsulated in the central part of the hydrophobic cavity, part of the MD run is near the primary rim, and part of the simulation is near the secondary rim, thus showing freedom of motion in the formed inclusion complexes in a 1:1 stoichiometry (see the animations of the four MD runs provided in SI, Video S1). Then, the inclusion process occurred through both the wider secondary rim and the narrower primary rim, as found in our previous work that considered the implicit solvent [78].

The geometries representative of the inclusion process obtained after the MD runs with the drug closer to the primary rim (see Figure 1a) or to the secondary rim (see Figure 1b) or in the central part of the hydrophobic β-CD cavity (see Figure 1c,d) without water molecules, omitted for clarity, are shown in Figure 1 in a side view.

From an energy point of view, the inclusion process of the hydrophobic 5-FU drug in the β-CD cavity in both MD runs, performed at 300 K, occurred at the beginning of the MD runs. In general, in all MD runs, an initial decrease in the potential energy within 200 ps and fluctuations in an average value for the remaining simulation time were observed. In particular, in the initial stage, the Coulomb energy decreased within 200 ps, and the van der Waals contributions fluctuated around an average value during the MD runs, starting from the four different initial geometries considered.

**Figure 2:**
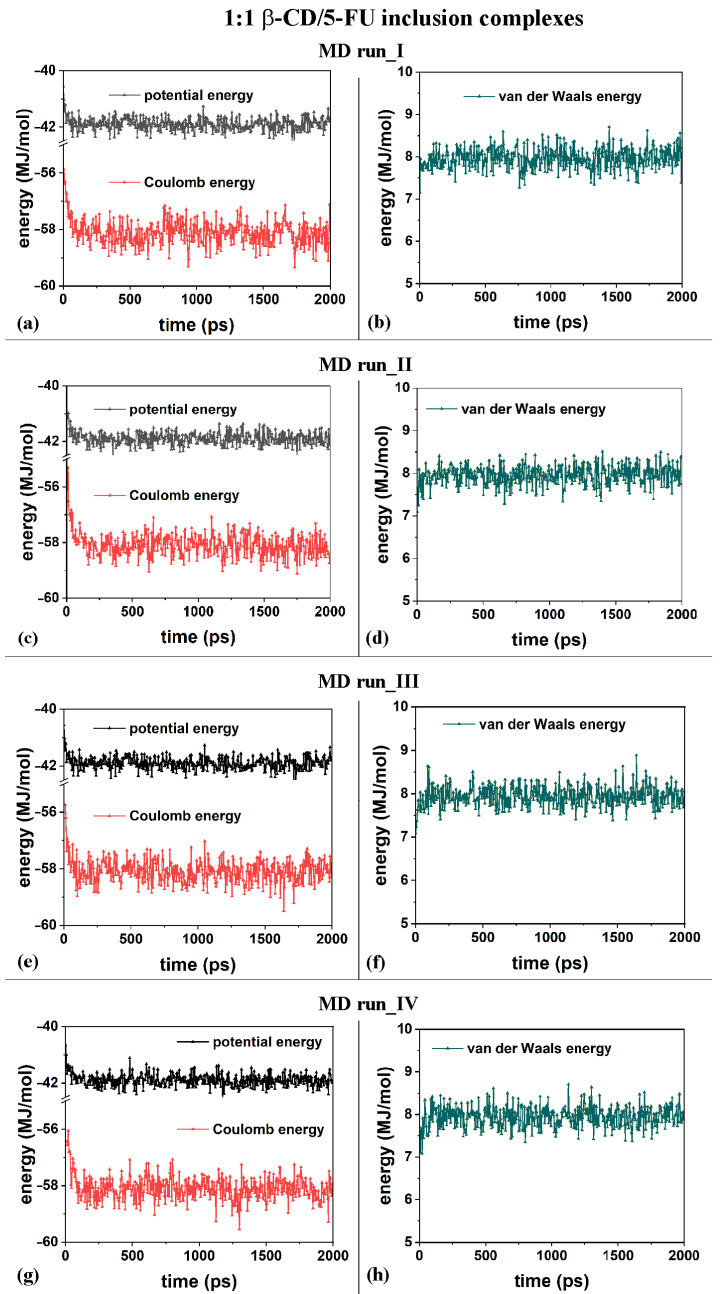
Panels (**a**,**c**,**e**,**g**) show the potential energy and Coulomb contributions; panels (**b**,**d**,**f**,**h**) show van der Waals energy calculated during the four MD runs performed, which lasted for 2 ns, in water, starting with the 5-FU drug molecule initially located near the primary or secondary β-CD rim (see nonoptimized initial geometries in Figure S2).

The first MD run started with the nonoptimized initial geometry presented in Figure S1a (MD run_I), with the 5-FU molecule initially located close to the β-CD’s primary rim, parallel to it. The second MD run began with the nonoptimized initial geometry presented in Figure S1b (MD run_II), with the drug molecule initially close to the β-CD’s secondary rim, parallel to it. Similar initial geometries are considered with the drug near the primary or secondary rim but perpendicular to them; see Figure S1c and Figure S1d, respectively. Figure 2a,c,e,g show the potential and Coulomb energy, and Figure 2b,d,f,h show the van der Waals contributions calculated during the four MD simulations performed.

This hydrophobic drug is held in the hydrophobic cavity thanks to the favorable van der Waals interactions in water, which are also exhibited in our MD study using the distance-dependent dielectric constant of water and the formation of H-bonds with β-CD atoms [78]. Some of the H-bonds between the hydrogen in the -OH groups of the β-CD and the oxygen atoms in the 5-FU’s carboxylic groups, as well as some of the H-bonds between the oxygen in the -OH groups of the β-CD and the hydrogen atoms bonded to the nitrogen in the 5-FU’s structure, formed and were disrupted dynamically during the MD runs.

Interestingly, the complexes were formed and stabilized as a result of favorable intermolecular interactions with water molecules. The initial water molecules in the cavity of the β-CD emerged during the drug inclusion process, as found in a previous work (see Figure 9 in Reference [79]), confirming the entropic gain due to the expulsion of water molecules from the β-CD cavity and inclusion of only one molecule.

Concerning the complex’s stabilization over time, the water molecules after the inclusion process stabilized the formed host–guest complex thanks to the H-bonds between the 5-FU atoms and the water molecules in the CD cavity or those near the primary and secondary rims [78,79], as well as those in the cavity when the drug approached one of the two β-CD rims. In fact, these are the water molecules that hydrated the hydrophilic cyclodextrin’s surface and which, during the MD run, moved from the surface to the inner cavity or from the inner cavity to the primary and secondary CD rims due to a kinetic energy of 300 K. These water molecules near the hydrophobic cavity in this 1:1 inclusion complex interacted with the included 5-FU drug atoms, forming weak H-bonds that stabilized them.

**Figure 3:**
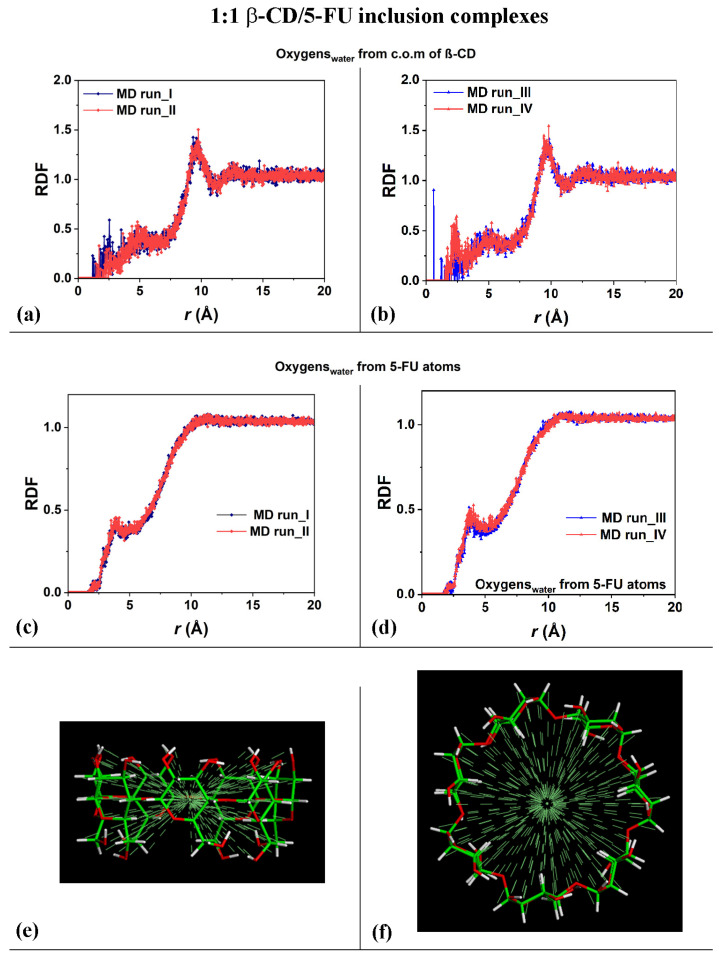
Panels (**a**,**b**): Plot showing the RDFs of the oxygen atoms of the water molecules as a function of the distance, r (in Å), from the c.o.m. of the β-CD calculated during the four MD runs performed. Panels (**c**,**d**): Plot showing the RDFs of the oxygen atoms of the water molecules as a function of the distance, r (in Å), from the 5-FU atoms calculated during the four MD runs performed. Panels (**e**,**f**): side view and top view of β-CD and its c.o.m. The color code is the same as that used in Figure 1.

In order to study the hydration of the β-CD/5-FU inclusion complexes in a 1:1 stoichiometry, the distribution of the water molecules around the host–guest complexes were investigated. The Radial Distribution Functions (RDFs) of the water molecules near the two 1:1 inclusion complex hosts and near the guest were calculated. In terms of the hydration of the β-CD, Figure 3a,b show the RDFs of the oxygen atoms of the water molecules as a function of the distance, *r*, from the center of mass (c.o.m.) of the β-CD calculated for the four MD runs that were performed. The side view and top view of the β-CD and its c.o.m. are reported in Figure 3e,f, for clarity.

The calculated RDFs have very similar values for both of the cases studied, but they do not have exactly the same hydration as that occurring in different MD runs. The sharp peaks centered at 9.61 Å and 9.75 Å in Figure 3a and at 10.0 Å and 9.81 Å in Figure 3b indicate the high probability density of finding the oxygen atoms of the water molecules around the β-CD c.o.m.; these distances obtained during the MD runs are most likely due to the first, most-ordered shell of hydration around the external surface of the β-CD. The broadest peaks at approximately 12.0 Å to 14.0 Å are due to the second, less-ordered shell of the water molecules around the outer β-CD surface. These are typical hydration shells that recall those already calculated for the native β-CD studied separately in water, as well as, in general, around hydrophilic material surfaces [78,79,90]. Interestingly, the oxygen in the water molecules also displayed a high probability density of being close to the β-CD c.o.m., from 1.17 Å to approximately 3.50 Å, and a second-highest probability density from 3.50 Å to 6.50 Å considering the first and the second MD run performed. Interestingly, a higher probability density of finding water molecules in β-CD is calculated in the third MD run at 0.59 Å (sharp peak) and in the fourth MD run at 2.23 Å, which garnered the relatively broadest peaks. These peaks are due to the water molecules near the CD cavity during the MD runs, due to the high mobility of this small drug when encapsulated. The β-CD interacts with the included 5-FU molecule and displays an interesting flexibility, always accommodating the moving of this small guest within it.

Figure 3c,d show the RDFs of the oxygen atoms of the water molecules as a function of the distance from the 5-FU atoms calculated for the four MD runs performed. The distribution of water molecules was very similar for both of the MD simulations. From 1.65 Å to 2.55 Å, the oxygen atoms in the water molecules were located near the included drug, and the sharper peak centered at approximately 3.89 Å indicates a second shell of water molecules around the 5-FU. The depletion from 5 to 10 Å is due to the space occupied by the cyclodextrin atoms, which display freedom of movement due to the kinetic energy at room temperature and its flexibility.

**Figure 4:**
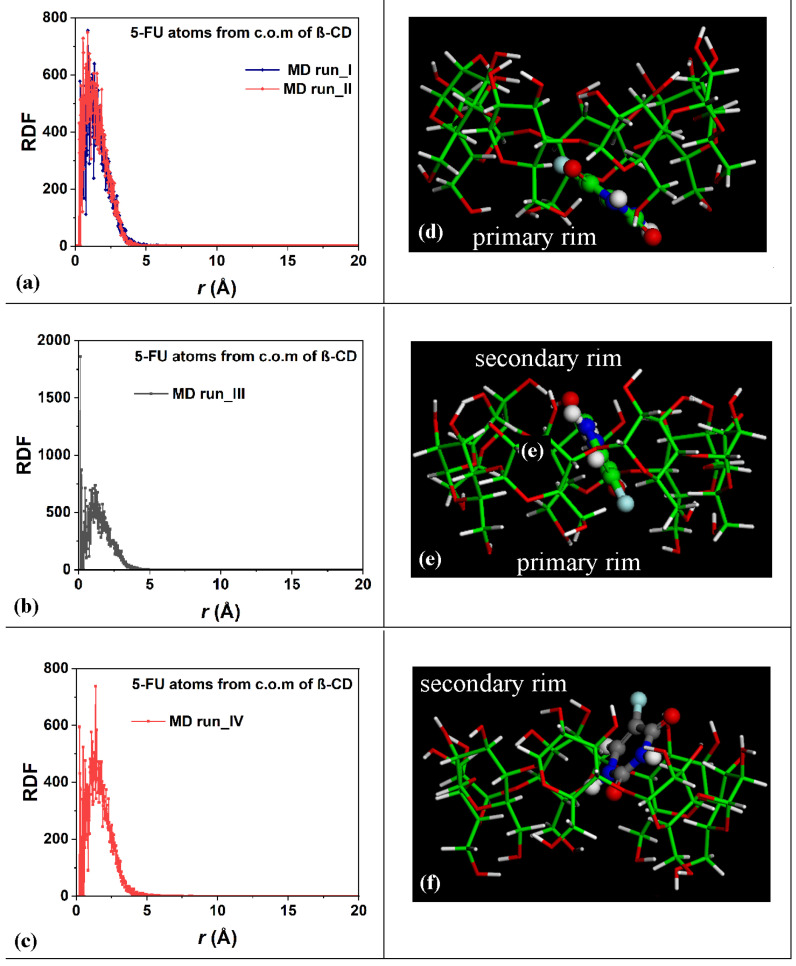
Panels (**a**–**c**): Plot showing the RDFs of the 5-FU atoms as a function of the distance, r (in Å), from the c.o.m. of the β-CD calculated for the four MD runs performed. Panels (**d**–**f**): nonoptimized instantaneous frames saved during the MD runs in which the 5-FU drug molecule is closer to the primary (panel (**d**)), in the central hydrophobic β-CD cavity (panel (**e**)), and closer to the secondary rim, respectively. The color code is the same as in Figure 1. All β-CD atoms are represented by sticks; all 5-FU atoms are represented by balls and sticks.

To study the distribution of the 5-FU drug atoms in the host cavity over time during the four MD runs performed, the RDFs of the drug atoms from the β-CD c.o.m. were calculated for the first and second MD runs, as shown in Figure 4a; for the third MD run, as shown in Figure 4b; and for the fourth MD run, as shown in Figure 4c.

Within 5 Å, the 5-FU molecule moved into the hydrophobic β-CD cavity. These data confirm the stability of the inclusion complexes formed during the two MD runs and the freedom of motion of the drug in the host cavity at room temperature in water. Figure 4d–f represent three different instantaneous nonoptimized geometries populated during the MD runs in which the 5-FU molecule was closer to the primary, in the central hydrophobic β-CD cavity, or closer to the secondary rim, respectively. Interestingly, similar geometries have been found in a dielectric medium (see Figure 1 in Reference [78]).

During the third MD run, the complexes periodically saved every 5 ps, with the 5-FU atoms for part of the simulation in the central part of the β-CD cavity, were populated; the distance between the 5-FU atoms from the β-CD c.o.m. equal to 0.13 Å indicates a high probability density of finding the drug in the hydrophobic cavity, as in the optimized geometry reported in Figure 1c and in the nonoptimized instantaneous conformation in Figure 4e, as a representative conformation of numerous geometries saved every 5 ps and studied during MD runs with this arrangement of the guest.

To elucidate all possible geometries of inclusion complexes of β-CD/5-FU in a 1:1 stoichiometry, an analysis of all conformations saved every 5 ps of the four MD runs was performed. In general, two possible similar geometries of interactions between β-CD and 5-FU, as reported in Figure 1, remained stable and populated over time. The guest molecule was observed in the β-CD cavity, near the primary rim, closer to the secondary rim or in the central part of the β-CD cavity. However, it is important to underline the fact that there is always a continuous equilibrium between part of the drug which is well included and part which is further away from the center of mass of the cyclodextrin, closer to the two different β-CD rims.

**Figure 5:**
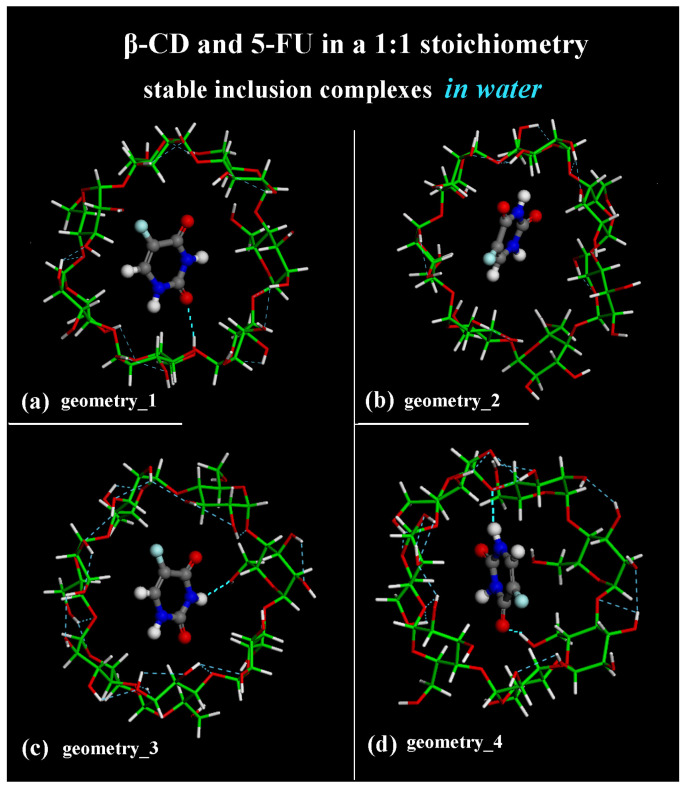
Top view of the optimized geometries after four MD runs, lasting for 2 ns, in water, with the 5-FU initially located near the primary rim or the secondary rim (see Figure S1a,c and Figure S1b,d, respectively). Cyclodextrin atoms are represented by sticks; 5-FU atoms are represented by balls and sticks. Color code: β-CD carbon atoms are indicated in green; carbon atoms of 5-FU in gray; oxygen in red; nitrogen in blue; fluorine in light blue; hydrogen in white. Water molecules are omitted for clarity.

The difference between these inclusion complexes populated during the MD runs performed can also be characterized by the intermolecular H-bonds formed between the β-CD and 5-FU in the hydrophobic cavity in the four optimized geometries after four MD runs in water (see Figure 5), in the final optimized geometries previously reported in side view in Figure 1.

In fact, the optimized geometry in Figure 1a after the first MD run shows an intermolecular H-bond between -C=O_5-FU_····H-O_primary rim of β-CD_: the calculated distance between the oxygen atom and the hydrogen is equal to 2.350 Å. The β-CD forms a total of 12 intramolecular H-bonds. In the optimized geometry in Figure 1b after the second MD run, there is no H-bond between the host and guest molecule; the β-CD forms a total of 11 intramolecular H-bonds. The optimized geometry in Figure 5c after the third MD run shows one intermolecular H-bond between -N―H_5-FU_····OH_primary rim of β-CD_: the calculated distance between the hydrogen atom and the oxygen is equal to 2.480 Å. The β-CD forms 18 intramolecular H-bonds. The optimized geometry in Figure 5d after the fourth MD run displays two intermolecular H-bonds between -C=O_5-FU_····H-O_primary rim of β-CD_ (distance calculated equal to 2.290 Å) and between -N―H_5-FU_····O_glycosidic of β-CD_ (distance equal to 2.367 Å). In this geometry, the β-CD forms 15 intramolecular H-bonds. The β-CD shows this interesting possibility of hosting hydrophobic small molecules within its cavity; however, at the same time, it can interact with it by dynamically forming and breaking H-bonds.

Melnikova et al. [84] identified the two most promising marker bands that could be used to detect complex formations: the C=O and C―F stretching bands of 5-FU, which experience a blue shift by ca. 8 and 2 cm^−1^ upon complexation. In these four optimized geometries reported in Figure 1 and Figure 5, studied in water, in particular, the -C=O group and -N―H atoms dynamically form H-bonds with the β-CD structure in the host–guest in a 1:1 stoichiometry. The intermolecular interaction involving -C―F atoms makes the β-CD/5-FU complexes particularly interesting.

**Figure 6:**
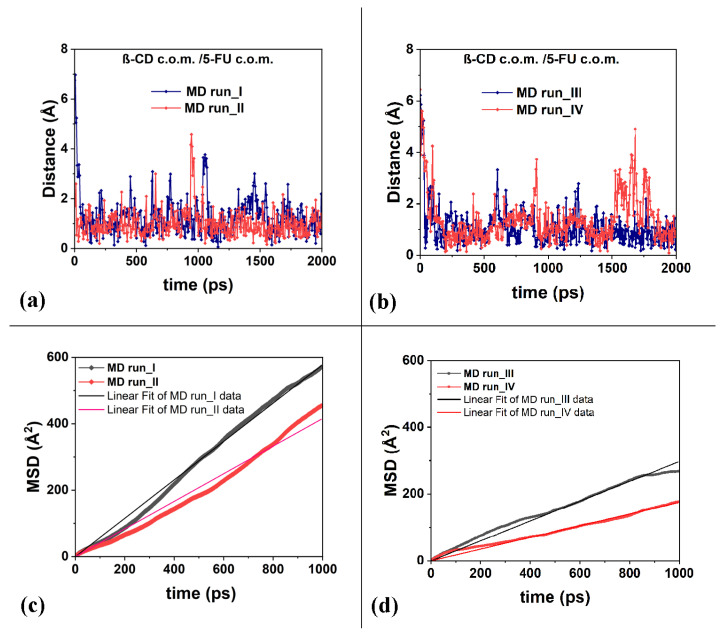
Panels (**a**,**b**): the distances between the c.o.m. of the 5-FU atoms and the c.o.m. of the β-CD calculated for the four MD runs considered. Panels (**c**,**d**): the mean square displacements (in Å^2^) related to the 5-FU drug molecule as a function of the time and the best linear fit lines that pass through the origin of the Cartesian axes calculated for the four runs studied, calculated from 0 to 1000 ps.

Thanks to its small size, the 5-FU molecule undergoes quite large fluctuations near the host cavity, as also shown by the distances between the c.o.m. of the 5-FU and the β-CD c.o.m. calculated over time during the four MD runs performed, as provided in Figure 6a,b. The short half-life of the β-CD/5-FU complex reported in the literature by Melnikova et al. [84] is likely related to the large mobility of this drug in the β-CD cavity.

To better characterize the freedom of motion of the 5-FU drug, its mean square displacements (MSDs) were calculated during the MD runs performed at 300 K and reported in Figure 6c,d. Data on the diffusion coefficient, *D*, calculated for the four MD runs from 0 to 1000 ps, are reported in Table 1.

It is interesting to note that the diffusion coefficients, *D*, calculated and reported in Table 1, are similar in order of magnitude to the self-diffusion coefficients of the chemically diverse pure liquid calculated using the all-atom molecular dynamics simulations reported by Baba et al. [91]. The calculations reported in this work, as well as in the literature, are in agreement with the experimental data. The authors suggest that MD calculations can be used as an excellent industrial tool for predicting, for example, molecular transportation in liquids, such as the diffusion of active ingredients in biological and pharmaceutical liquids. The mobility of the 5-FU drug in the hydrophobic cavity is high, and in some instantaneous frames saved during MD runs, it is relatively far from the cavity. This fact affects the different diffusion coefficient from 3.0 × 10^−10^ to 9.6 × 10^−10^ (m^2^/s).

In this section of the present work, MD simulations in water are used to explain the formation and hydration of host–guest complexes and the possible diffusion in water for a better understanding, at the atomistic level, of the drug release mechanism in a biological environment. Because of its small size, the 5-FU molecule suggests the possibility of forming β-CD inclusion complexes in a 1:2 stoichiometry, as found in a previous work using the distance-dependent dielectric constant of water [78] and discussed in the next section.

### 2.2. β-CD/5-FU Inclusion Complexes in a 1:2 Host–Guest Stoichiometry in Water

The interaction between the β-CD and 5-FU drug molecules in 1:2 host–guest stoichiometry in water in a simulation cell with periodic boundary conditions was studied without assuming any a priori inclusion complexes [30,78,79]. Four initial different geometries were considered with the two 5-FU drug molecules located far from the β-CD cavities, in particular, in the first and third MD runs initially close to the primary β-CD rim in a perpendicular and parallel arrangement, as outlined in Figure S2a and Figure S2c, respectively, and in the second and fourth MD runs close to the secondary rim in a parallel and perpendicular arrangement, as outlined in Figure S2b and Figure S2d, respectively.

After the initial energy minimizations, the MD runs, which lasted for 2 ns, at 300 K, analyzing all configurations assumed by the system saved every 5 ps during MD runs, and the optimizations of the final geometries assumed by the systems when an equilibrium state was achieved, the drug inclusion process occurred across both rims (see the animations of the four MD runs provided in the SI, Video S2). Interestingly, the more populated inclusion geometries are very similar to two different arrangements that were found in a previous study performed using the distance-dependent dielectric constant of water (see Figure 2 in Reference [78]). In water, the mobility of drugs is greater. This is evident in the β-CD/5-FU inclusion complexes in a 1:1 stoichiometry, where instances of the drug being freely distant from β-CD are observed in populated and instantaneous frames saved every 5 ps.

**Figure 7:**
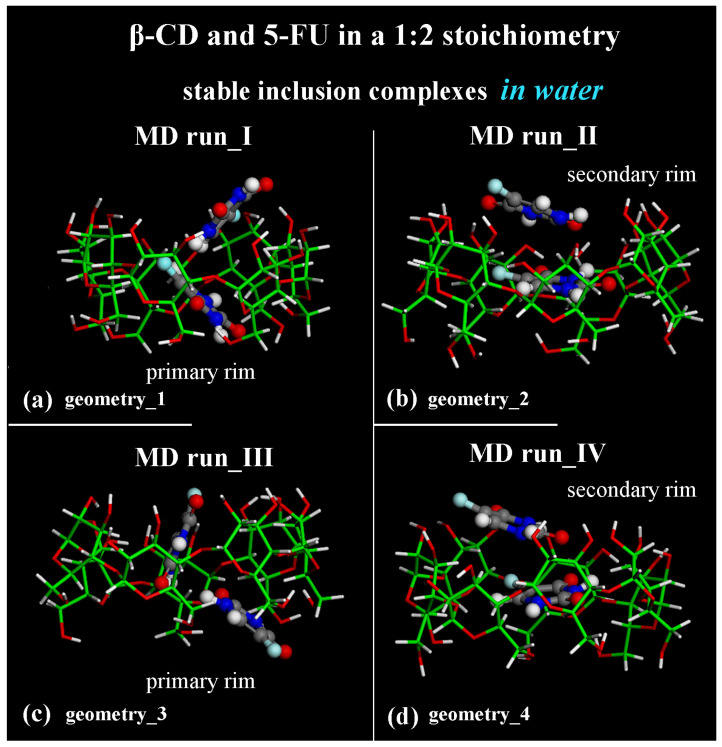
Side view of the final optimized geometries after the four different MD runs, lasting for 2 ns, in water, with the two 5-FU drug molecules initially located close to the primary rim (see Figure S2a,c) or close to the secondary rim (see Figure S2b,d). The color code is the same as in Figure 1. Water molecules are omitted for clarity.

Considering the optimized geometries at the end of the four MD runs performed, after the first MD run, the final optimized geometry displayed the two 5-FU drug molecules tilted by approximately 90° toward each other (see Figure 7a); in the second MD run performed, the drug molecules were almost parallel in the hydrophobic β-CD cavity (see Figure 7b). In both cases, one drug molecule is buried in the CD cavity, while the other one is closer to the secondary rim, interacting with the former. In Figure 7, water molecules are omitted for clarity.

Adopting the same procedure proposed for the study of possible inclusion complexes in a 1:1 stoichiometry, two MD runs are also subsequently performed, considering the 5-FU drugs initially located close to the β-CD’s primary or secondary rim, parallel or perpendicular to them, as reported in Figure S2c,d. Even considering these additional MD runs, only these two different host–guest inclusion complexes were found with the drug near the primary or secondary rim, as reported in two previous MD runs (see Figure 7c,d). In fact, by analyzing all conformations assumed by the system during these additional MD runs saved every 5 ps, the two possible geometries of interactions between β-CD and 5-FU are stable and populate over time. They display the 5-FU drugs encapsulated in two distinct orientations: tilted by approximately 90° toward each other (compare Figure 7a,c) or almost parallel in the hydrophobic β-CD cavity (compare Figure 7b,d).

**Figure 8:**
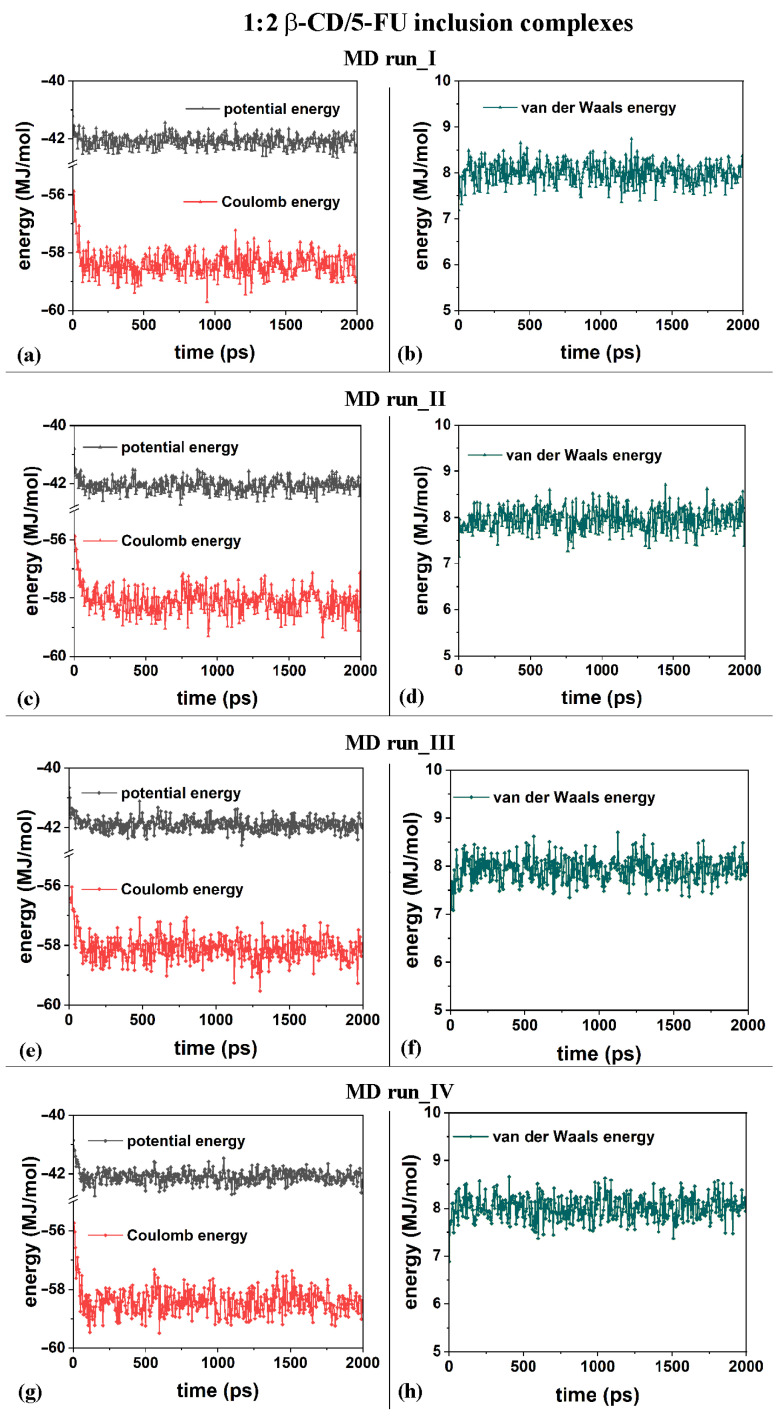
Panels (**a**,**c**,**e**,**g**): the potential energy and Coulomb contributions; panels (**b**,**d**,**f**,**h**): van der Waals energy calculated for the four MD runs performed, lasting for 2 ns, in water, with the two 5-FU drug molecules initially located near the primary or secondary β-CD rim (see nonoptimized initial geometries in Figure S2).

From an energetic point of view, during the four different MD runs performed at 300 K in water, and considering the β-CD and 5-FU in a 1:2 stoichiometry, the potential energy, as well as the Coulomb energy, initially decreased, and the van der Waals contributions fluctuated around an average value (see Figure 8a–h), as was found and discussed regarding the MD simulations considering β-CD/5-FU in a 1:1 stoichiometry. The inclusion process of the 5-FU drugs in the hydrophobic β-CD cavities occurred rapidly (see all animations for the four MD runs in Video S2).

**Figure 9:**
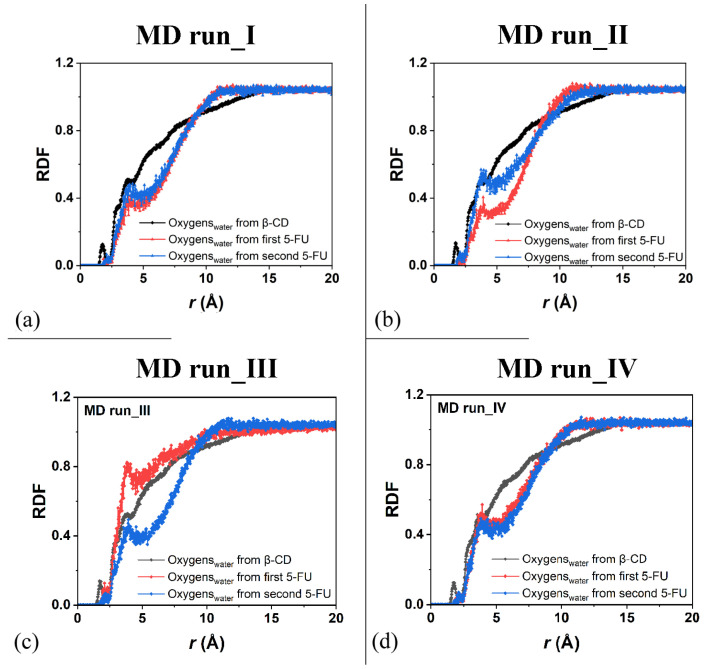
Panels (**a**–**d**): the RDFs of the oxygen atoms of the water molecules calculated for the four MD runs performed as a function of the distance, r (in Å), from the β-CD atoms (black) and from the 5-FU atoms of the two drug molecules (red and blue, respectively).

Concerning the hydration of the β-CD in the inclusion complexes in a 1:2 host–guest stoichiometry during the first MD run, Figure 9a displays the RDFs of the oxygen atoms of the water molecules around all of the β-CD atoms (black), with a higher probability density of finding them centered at approximately 1.77, 2.85, and 3.73 Å, as for typical hydrated hydrophilic surfaces [79,80].

For the hydration of the 5-FU molecules in the inclusion complexes, Figure 9a displays the RDFs of the oxygen atoms of the water molecules around the first 5-FU (red) and second (blue) molecules, indicating relatively greater hydration during the MD simulations because they were more exposed to water molecules. Figure 9b shows the same RDFs calculated for the second MD run, at the end of which the drug molecules are, on average, parallel to each other. The hydration of all of the β-CD atoms was exactly the same; however, the water’s distribution around the two 5-FU molecules was different because they moved differently in two different environments.

The drug that was included to a greater extent in the hydrophobic cavity moved more toward the primary rim, which is the relatively narrower one, and it was less hydrated. The drug molecule closest to the secondary rim, the relatively wider rim, was both more mobile and more hydrated (see the sharper peaks centered at approximately 2.0 and 3.95 Å; line and symbols in blue). Similar hydration of the β-CD and drugs is observed in the other two additional MD runs showing similar host–guest complexes with some important differences due to the freedom of motion of the 5-FU molecule. During MD run III, the second 5-FU molecule moves far from the β-CD cavity and the well-hydrated molecules moves in the simulation cell; its hydration is then higher than the second drug encapsulated in the hydrophobic cavity (see Figure 9c).

Interestingly, when the two 5-FU molecules are included in the β-CD cavity and tilted by approximately 90° toward each other with the second 5-FU more exposed from the primary rim, the β-CD primary rim is relatively larger than the secondary one, both in the geometry optimized after the MD run and in numerous instantaneous conformations periodically saved during the MD run. When the two drugs are, on average, parallel in the CD cavity, the hydration of the drug molecule is very similar, as reported in Figure 9d.

Dynamically, during the MD runs, the β-CD shows flexibility and periodic motion of the β-CD glucose unit, as found in our previous theoretical study [92]. During MD runs in water, β-CD shows flexibility by hosting the small guest drug with freedom of movement. On average, the secondary edge tends to be wider over time. However, following the movement of the guest molecule during MD runs, the primary rim may appear larger in the instantaneous frame periodically saved, due to the flexibility of the cyclodextrin at room temperature and the tendency to maximize the intermolecular interactions with the encapsulated guest molecule.

**Figure 10:**
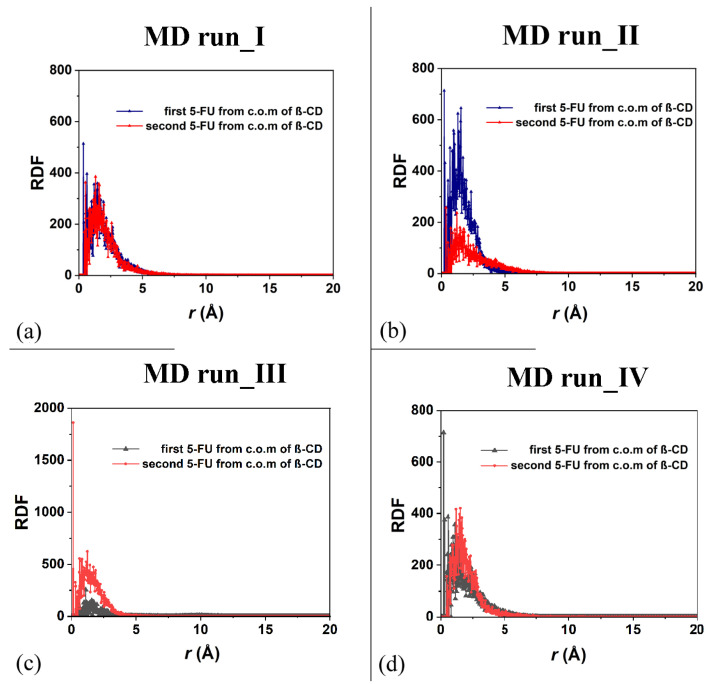
Panels (**a**,**b**): the RDF of the first and second 5-FU molecules (blue and red lines and symbols, respectively) as a function of the distance, r (in Å), from the c.o.m. of β-CD calculated during the first and the second MD runs performed. Panels (**c**,**d**): the RDF of the first and second 5-FU molecules (black and red lines and symbols, respectively) as a function of the distance, r (in Å), from the c.o.m. of β-CD calculated during the first, third and fourth MD runs performed.

As was carried out for the 1:1 host–guest complex, the RDFs of the drug atoms calculated relative to the β-CD c.o.m. are shown in Figure 10. While in the first MD run, the two drug molecules displayed similar degrees of hydration and motion related to the β-CD c.o.m., in the second one, both the hydration and the motion of the two 5-FU drugs were different: one molecule was, on average, included to a greater extent in the cavity, closer to the primary rim, and less exposed to the solvent; the second one was included to a lesser extent, was closer to the secondary edge, and, on average, wider and more well hydrated. During the third MD run, the first molecule moved far from the cavity; thus, the probability density of finding its atoms from the β-CD c.o.m. was smaller than for the second one, which was well encapsulated during the MD run for numerous instantaneous conformations assumed saved every 5 ps. The sharp peak centered at 0.13 Å is due to this well-encapsulated drug. During the fourth MD run, the two 5-FU molecules, almost parallel, demonstrate that the first drug is more encapsulated and that the second one is more exposed to the water molecules around the inclusion complexes.

In order to study the relative motion of the two drugs during MD simulations, particularly the distance between drug molecules during the first and the second MD runs, which display two different kinds of inclusion complexes over time (see Figure 7a,b), Figure S3a shows the distances between the two c.o.m.s of the 5-FU molecules calculated for the MD runs. In the first MD run, the maximum distance between the two centers of mass was, in fact, 11.3 Å (black line and symbols). The instantaneous configuration assumed by the system at 860 ps, the time to which the calculated maximum distance corresponds, is reported in Figure S3b. The distance between the hydrogen in the -CH group included in the hydrophobic cavity and the c.o.m. of the six-membered ring of the second 5-FU was equal to 2.698 Å. The molecules seem to be distant, but with weak interactions taking place.

During the second MD run, the maximum distance between the two centers of mass was equal to 8.26 Å (green line and symbols in Figure S3a). The instantaneous configuration assumed by the system at 1685 ps, the time to which the calculated maximum distance corresponds, is reported in Figure S3c. Interestingly, the distance between the hydrogen in the -CH group included in the hydrophobic cavity and the fluorine atoms of the second 5-FU molecule was equal 3.169 Å. Again, the two 5-FU drug molecules seem to be distant, but with weak interactions taking place. It is interesting to note that this possibility of intermolecular interactions between the fluorine atoms in the 5-FU structure and the -CH groups could be important when these inclusion complexes which are formed are near the plasmatic membrane. The release across the membrane can take place thanks to both the freedom of motion of the drug molecules in host–guest complexes and the good intermolecular interactions due to the fluorine atoms in the drug’s structure.

**Figure 11:**
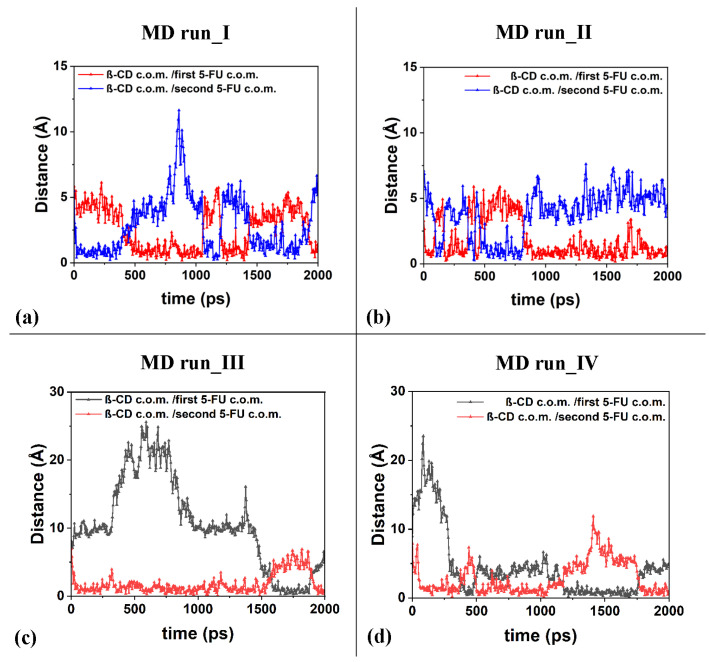
Panels (**a**–**d**): the distances between the c.o.m.s of the two different 5-FU molecules and the c.o.m.s of the β-CD calculated for the four MD runs performed.

The freedom of motion of the two included 5-FU molecules in the β-CD is correlated: when one molecule approaches the hydrophobic cavity, the other moves away, while maintaining a distance that allows for favorable intermolecular interactions. It is important to underline the fact that in the four molecular dynamics simulations, the interaction geometries of the host–guest complexes with the 5-FU molecules partly parallel to each other and partly perpendicular are populated. Figure 11 shows the distances between the c.o.m.s of the two different 5-FU molecules and the c.o.m.s of the β-CD calculated during the four MD runs.

Interestingly, during the third MD performed for part of the time, only the 1:1 inclusion complex is present in the simulation cell, as a hydrated 5-FU molecule moves away from the β-CD. This suggests that the lifetime of the 1:2 complex may decrease over time, when, in fact, only one molecule remains included. In the other three dynamics performed, the 1:2 complex is more populated. In these three simulations, observing Figure 11a–d, the anti-correlation present in the variation in the distances calculated during MD runs between the c.o.m. of the drug molecules and the c.o.m. of the β-CD is particularly interesting at short distances. This may be due to the fact that two 5-FU molecules, albeit small, do not occupy the same space, so the confined motion of the two small 5-FU molecules is anti-correlated in the β-CD cavity; that is, when a drug molecule can move closer to the center of mass of the β-CD, the other one moves more towards the primary or secondary edge. The motion of the two drug molecules in a confined space is anti-correlated.

**Figure 12:**
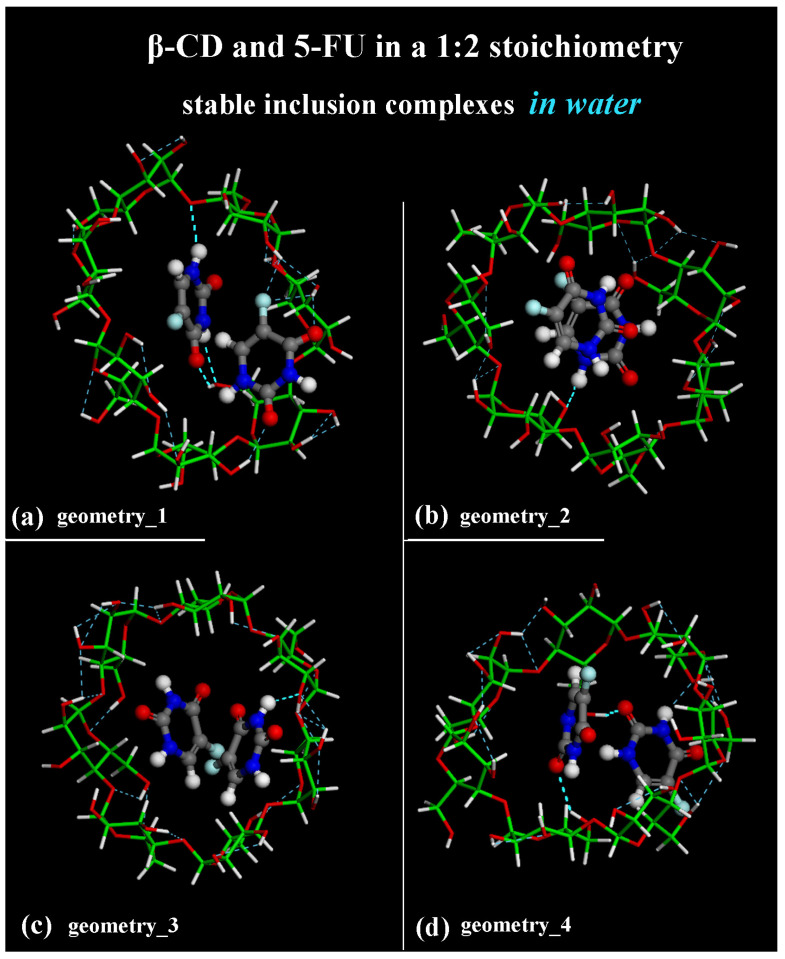
Panels (**a**–**d**): top view of the same optimized geometries reported in Figure 7 after the four different MD runs, lasting for 2 ns, in water, considering β-CD and two 5-FU in a simulation cell. The color code is the same as in Figure 7. Water molecules are omitted for clarity.

The difference between the inclusion complexes populated during the four MD runs performed for the host–guest complexes in a 1:2 stoichiometry can be characterized as previously, by the intermolecular H-bonds between the β-CD and two 5-FU molecules, as reported in Figure 12.

In the optimized geometries obtained at the end of MD runs, the same geometries reported in the side view in Figure 7, now shown in top view, are studied.

In Figure 12a, the more encapsulated 5-FU drug forms two H-bonds involving -N―H_5-FU_····O_primary hydroxyl group of β-CD_ (distance: 2.370 Å) and -C=O_5-FU_····H―O_primary rim of β-CD_ (distance: 1.971 Å). In Figure 12a, the second 5-FU molecule closer to the secondary rim forms three H-bonds involving -C=O_5-FU_····H―O_secondary rim of β-CD_ (distance: 2.402 Å), -C―F_5-FU_····H―O_secondary rim of β-CD_ (distance: 2.393 Å) and -C―F_5-FU_····H―O_secondary rim of β-CD_ (distance: 2.289 Å).

In Figure 12b, the more encapsulated 5-FU drug forms one H-bond involving -N―H_5-FU_····O_primary hydroxyl group of β-CD_ (distance: 2.331 Å). 

In the optimized geometry in Figure 12c, only the 5-FU molecule closer to the secondary rim more exposed to the hydration forms one H-bond involving -N―H_5-FU_····O_secondary hydroxyl group of β-CD_ (distance: 2.355 Å).

In the inclusion complex in Figure 12d, the 5-FU closer to the primary rim forms an H-bond involving -C=O_5-FU_····H―O_primary rim of β-CD_ (distance: 2.186 Å); the 5-FU closer to the secondary rim forms an H-bond involving -C=O_5-FU_····H―O_secondary rim of β-CD_ (distance: 2.498 Å). It is interesting to highlight that these intermolecular interactions form and break dynamically during the MD simulations of all four MD runs performed. These are just four different optimized geometries populated over time in all conformations assumed by the system to be saved every 5 ps during the simulations in water. Again, as suggested and discussed by Melnikova et al. [84], the C=O and C―F stretching bands can be useful for studying the complexation process. A first small molecule of 5-FU can be easily encapsulated in the hydrophobic cavity of β-CD. Of interest is the freedom of movement of both the included 5-FU drug and the cyclodextrin, which can accommodate another small 5-FU thanks to intermolecular H-bonds and the weak van der Waals interaction between the cyclodextrin cavity and the first included guest.

To better characterize the freedom motion of the 5-FU drugs, their mean square displacements (MSDs) were calculated during the MD runs, at 300 K, as reported in Figure S4. The information for the diffusion coefficient, *D*, calculated for the MD runs from 0 to 300 ps considering the linear slop in all four MD runs performed, is reported in Table S1.

As for the 1:1 host–guest inclusion complexes, the diffusion coefficients, *D*, calculated and reported in Table S1, are similar in order of magnitude to the self-diffusion coefficients of the chemically diverse pure liquid calculated using the all-atom molecular dynamics simulations reported by Baba et al. [91]. It must be highlighted that the 5-FU molecule that diffuses in water in the first part of the third MD run shows a larger diffusion coefficient with respect to the other drug molecules in inclusion complexes studied or, over time, which are far from the β-CD cavity, closer to primary or secondary rims.

It is important to better understand the different drug mobilities in hydrophobic cavities of host–guest complexes between β-cyclodextrin and 5-fluorouracil at different stoichiometries over time, because aqueous solutions will have kinetic energy at specific temperatures, which can overcome the strength of interaction energies more significantly in a confined hydrophobic cavity and are affected by random dynamics of the collision in aqueous solutions.

### 2.3. β-CD and Two 5-FU Molecules in a Random Arrangement in Water: From 1:1 to 1:2 Inclusion Complex Formation with Different Stabilities

The interaction between the β-CD and two 5-FU drug molecules in an initial random arrangement in water was studied to verify the possibility of forming host–guest complexes, as in previous sections.

The initial nonoptimized geometry studied is reported in Figure S5. Using the same protocol as before, after energy minimization, in the MD run lasting for 10 ns at a constant temperature equal to 300 K in water in a similar simulation cell with periodic boundary conditions, the possible inclusion complexes were studied without assuming any a priori inclusion complexes [30,78,79] (see the animation of the MD run provided in the SI, Video S3). The conformations assumed by the systems were saved every 5 ps and analyzed at the end of the MD run. The animation file is reported in Video S3. In Figure S6, showing the potential energy, the Coulomb contributions are reported in panel (a); the van der Waals energy is reported in panel (b).

**Figure 13:**
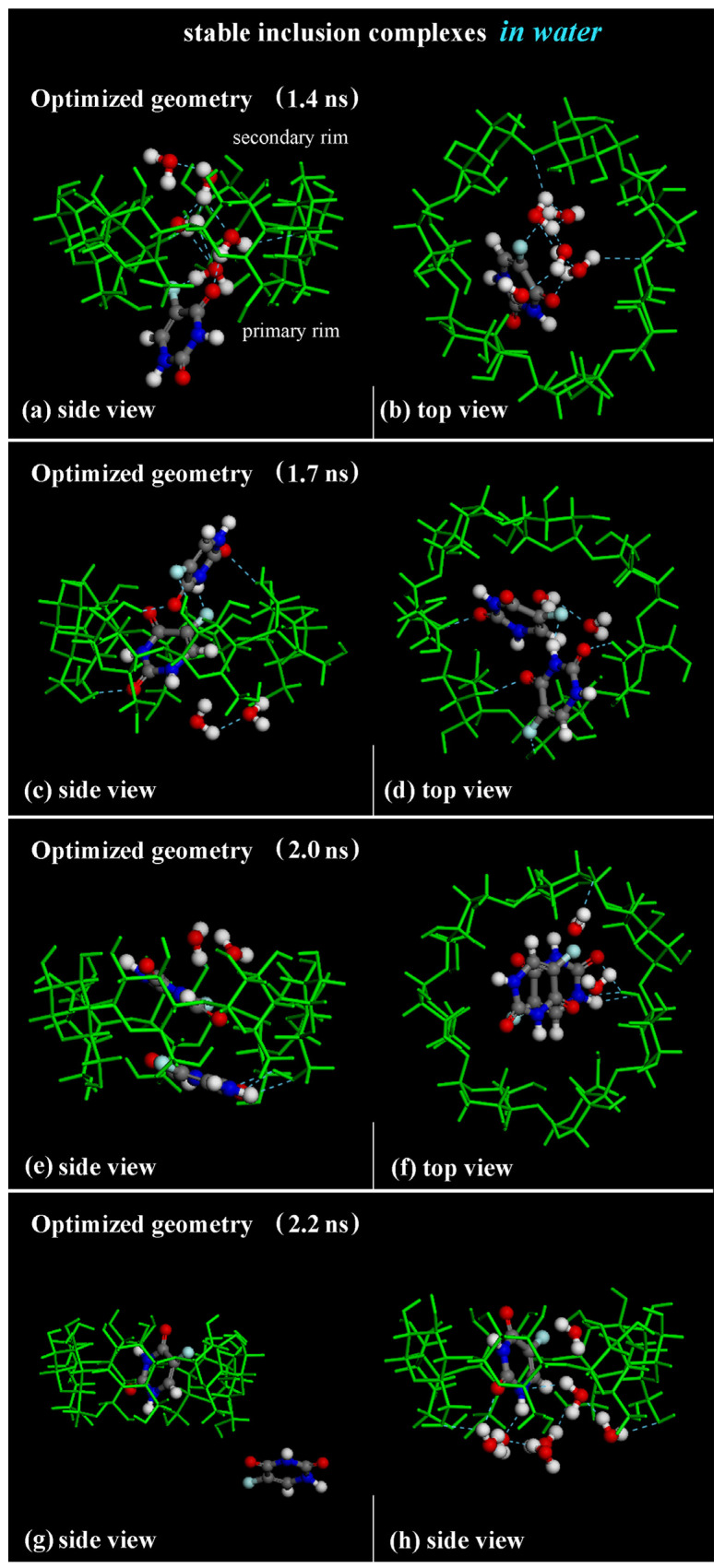
Panels (**a**–**f**): Side view and top view of the optimized geometries saved during MD run lasting for 10 ns in water at 1.4 ns, 1.7 ns and 2.0 ns, respectively. Panels (**g**,**h**): side view of 1:1 β-CD/5-FU inclusion complex and one 5-FU “free” drug in water, omitted for clarity. In all other panels, the hydration water molecules that form H-bonds near the hydrophobic cavity are reported. Color code: all β-CD atoms are indicated in green; carbon atoms of 5-FU in gray; oxygen in red; nitrogen in blue; fluorine in light blue; hydrogen in white.

As in previous MD runs performed and discussed in Section 2.1 and Section 2.2, at first, the 1:1 inclusion complex is formed and stable over time, inducing the expulsion of water molecules initially in the cavity of the β-CD, as found in previous theoretical work [51,79,80]. Figure 13a,b show the optimized geometry saved during the MD run at 1.4 ns. Water molecules in the β-CD cavity form H-bonds with a fluorine atom in the -C-F group and with the oxygen atom of -C=O groups.

The first 5-FU enters the β-CD cavity, initially forming the host–guest inclusion complex in a 1:1 stoichiometry. During the MD run, the inclusion complex β-CD/5-FU in a 1:2 stoichiometry is formed with two molecules almost perpendicular or parallel in the hydrophobic cavity, as found in previous MD runs performed and shown in the geometry optimized at 1.7 ns and 2.0 ns (see Figure 13c–f). When the drug molecules are encapsulated, water molecules tend to be close to the drug near the primary or secondary rim to effectively hydrate the inclusion complex, as found in previous sections.

While the 1:1 inclusion complex is stable, the 1:2 host–guest complexes are less stable, and during the simulation time, these complexes break down and reform the formation of a 1:1 host–guest complex occurring with one 5-FU molecule “free” in water, as reported, for example, in the optimized geometry saved at 2.2 ns in Figure 13g,h. A continuous equilibrium exists between the formation of the 1:2 inclusion complex and the resulting 1:1 inclusion complex, alongside the presence of a 5-FU “free” in water during the MD run. This observation confirms their varying stability over time: the complex with an encapsulated 5-FU is the most stable, but it becomes less stable when a second drug interacts with the hydrophobic cavity and the included 5-FU. These results are in agreement with experimental results presented in the literature [84].

## 3. Materials and Methods

The MM and MD simulations in water were performed by adopting the simulation protocol proposed in previous work [78,79] concerning the formation of host–guest inclusion complexes. No geometry of inclusion was assumed a priori; the eight initial trial geometries with 1 or 2 molecules of 5-FU were randomly placed outside the CD cavity near the primary and secondary rim. The system was fully hydrated in a cubic cell with one side equal to 33.6 Å considering the periodic boundary conditions. The simulation protocol consisted of three steps: (I) energy minimizations of the initial trial β-CD and 5-FU geometries with the drug molecule initially near the primary and secondary rims of the β-CD; (II) MD runs at T = 300 K until an equilibrium state was achieved, and analysis of the conformations assumed by the systems every 5 ps; and, (III) geometry optimizations of the system at the end of the MD runs and the studied instantaneous frames captured during the MD runs [78]. All calculations were performed using the Materials Studio package 7.0 (BIOVIA, [93]) and the CVFF force field [94]. All energy minimizations were carried out using the Conjugate Gradient algorithm up to an energy gradient lower than 4·10^−3^ kJ mol^−1^ Å^−1^. The stable inclusion complexes in both 1:1 and 1:2 stoichiometries were investigated. First, β-CD:5-FU in a 1:1 stoichiometry in four different initial geometries was studied, with the drug molecule initially located near to the primary or secondary rim (see Figure S1). Then, considering β-CD:5-FU in a 1:2 stoichiometry, four initial geometries were investigated with two 5-FUs near the primary or secondary rim (see Figure S2). Using a simulation protocol for the implicit solvent that was proposed in a previous work, after an initial energy minimization, MD runs lasting for 2 ns and the final geometry optimizations of all stable and metastable inclusion complexes were characterized. All MD simulations were performed at a constant temperature controlled through the Berendsen thermostat. The integration of the dynamical equations was carried out with the Verlet algorithm using a time step of 1 fs. The equilibration state was monitored by following the evolution of the potential energy and its components from an energetic point of view and following the evolution of the selected geometrical parameters over time, specifically the distance between the centers of mass (c.o.m.) of the β-CD and 5-FU and the radial distribution function (RDF) of the water oxygens from the CD center of mass for investigating the water distribution around the inclusion complexes formed in water. A ninth MD run considering the simulation cell with β-CD and two 5-FU molecules in a random arrangement was performed to study the possibility of forming an inclusion complex in a 1:1 stoichiometry and/or in a 1:2 stoichiometry lasting for 10 ns in water and their stability over time.

## 4. Conclusions

In this theoretical work, β-CD and the anticancer drug 5-FU were investigated in water in both 1:1 and 1:2 stoichiometries. The 5-FU drug is a small molecule, allowing both the first and second drug molecules to be included in the hydrophobic β-CD cavity. The 1:1 inclusion complexes formed in water are stable over time. The 1:2 inclusion complexes are less stable over time, with a high freedom of movement of the second drug, which, when moving away from the β-CD cavity, explains the formation of only 1:1 host–guest complexes, in good agreement with experimental data [84].

In a 1:1 stoichiometry, the fluorine atom in the 5-FU structure is oriented toward the primary or secondary rim. In a 1:2 stoichiometry, one of the 5-FU molecules is either parallel or almost perpendicular to the first fully enclosed drug molecule, partially exposed to water molecules that hydrate the complex. This study confirms the importance of favorable intermolecular interactions between the β-CD and drugs in the carrier cavity, which are sometimes more exposed to the primary or secondary rim and therefore to hydration. At room temperature, a continuous balance between the van der Waals interactions, together with the possibility of forming some H-bonds in the hydrophobic β-CD cavity, occurs in the presence of water. This hydration process predominantly affects drug molecules that are more exposed to the surrounding water.

The 5-FU drugs interact not only because of hydrophobic interactions but also involving the -C=O, -N-H, -CH and -CF groups, confirming the importance of these interactions after complexation, as found by Melnikova et al. [84]. All weak intermolecular interactions affect the mobility and diffusion of drug molecules in the 1:2 complexes: a faster release for the 5-FU more exposed to the water molecules, and a slower release for the totally encapsulated ones. The large mobility of the drug in the β-CD structure likely explains the short lifetime experimentally measured [84].

Interestingly, in the 1:1 complexes studied, the diffusion coefficients calculated for the MD runs were similar to those calculated by Baba et al. in the literature [91], and they were larger on average than the same data calculated in 1:2 host–guest complexes. In instantaneous frames captured during an MD run, a 5-FU is relatively far from the CD, suggesting the possibility of its release in aqueous solution. This 5-FU molecule furthest from the β-CD cavity can be considered, as recognized by Petaccia et al., as “free 5-FU” [95,96], a more hydrated, more mobile molecule, suggesting a possible easy release in a biological environment. The bioavailability of 5-FU is connected to the freedom of movement that can be studied using MM and MD simulations, which can help in the investigation of the hydration of inclusion complexes, the possible different release processes due to the intermolecular interactions in a more- or less-hydrated environment, and the influence of the β-CD/drug stoichiometries in the diffusion coefficient of drugs in water, with interesting comparisons with experimental data [84]. The different time of release could be a key factor in permitting the release of drugs in a biological environment at different times, to avoid local side effects over time. Molecular dynamics simulations can help to better understand this aspect by combining theoretical investigation and comparison with experimental data [97,98,99].

Greater focus on not only the drug design process but also the design of new carriers could improve research and outcomes for the solubilization, transport, and diffusion of hydrophobic drugs in a biological environment. It is likely that, by using modified cyclodextrins [15,16] or cross-linked β-cyclodextrins in nanosponge systems [30], or the γ-CD which has a larger hydrophobic cavity [100], it will be possible to study a new innovative carrier for the solubilization of this anticancer.

## Figures and Tables

**Table 1:** Data on the slope of the best linear fit of the MSDs calculated for the four MD runs reported in Figure 6c,d, as well as the diffusion coefficient (*D*) and *R*^2^.

	**Slope (Å^2^/ps)**	***D* (m^2^/s)**	***R* ^2^**
MD run_I	0.57774 ± 0.00184	9.6290 × 10^−10^	0.99798
MD run_II	0.41579 ± 0.00256	6.9628 × 10^−10^	0.99254
MD run_III	0.29751 ± 0.00130	4.9585 × 10^−10^	0.99620
MD run_IV	0.17505 ± 0.00072	2.9175 × 10^−10^	0.99656

## Data Availability

The data presented in this study are available on request from the corresponding author.

## Supplementary Materials

The following supporting information can be downloaded at: https://www.mdpi.com/article/10.3390/ijms25115888/s1.
