# Supplementary material for: Different Drug Mobilities in Hydrophobic Cavities of Host–Guest Complexes between β-Cyclodextrin and 5-Fluorouracil at Different Stoichiometries: A Molecular Dynamics Study in Water
Source: Int J Mol Sci. 2024 May 28;25(11):5888. doi: 10.3390/ijms25115888 (PMC11172661; doi:10.3390/ijms25115888)
Supplement: Supplementary file 1 [file ijms-25-05888-s001.zip › ijms-3009227-supplementary.pdf]

International Journal of
Molecular Sciences

MDPI

Supplementary Materials

Different Drug Mobilities in Hydrophobic Cavities of Host–Guest Complexes between β-Cyclodextrin and 5-Fluorouracil at Different Stoichiometries: A Molecular Dynamics Study in Water

Giuseppina Raffaini [1,2,*], Stefano Elli [3], Michelina Catauro [4] and Antonio D'Angelo [4]

[1] Department of Chemistry, Materials, and Chemical Engineering "Giulio Natta", Politecnico di Milano, Piazza L. Da Vinci 32, 20131 Milano, Italy
[2] INSTM, National Consortium of Materials Science and Technology, Local Unit Politecnico di Milano, 50121 Milano, Italy
[3] Istituto di Ricerche Chimiche e Biochimiche 'G. Ronzoni', Via Giuseppe Colombo 81, 20133 Milano, Italy; elli@ronzoni.it
[4] Department of Engineering, University of Campania "Luigi Vanvitelli", Via Roma 29, 81031 Aversa, Italy; michelina.catauro@unicampania.it (M.C.); antonio.dangelo@unicampania.it (A.D.)
* Correspondence: giuseppina.raffaini@polimi.it

Supplementary Materials

2.1. β-CD/5-FU inclusion complexes in a 1:1 host–guest stoichiometry in water

Figure S1. Nonoptimized initial geometries before the four MD runs, lasting for 2 ns, in water, with the 5-FU initially located near the primary rim (see panels (a) and (c)) or near the secondary rim (see panels (b) and (d)). Color code: β-CD carbon atoms are represented by green sticks; carbon atoms of the 5-FU represented by balls and sticks in gray; oxygen in red; nitrogen in blue; fluorine in light blue; hydrogen in white. Water molecules are represented by sticks; oxygen in red; hydrogen in white…………………………S3

Video S1: The four MD runs obtained starting from the optimized geometries in Figure S1 are reported in file.avi generated using the Materials Studio program, visualizing the conformations saved every 5 ps during MD runs lasting for 2 ns………………..……..S3

2.1. β-CD/5-FU inclusion complexes in a 1:2 host–guest stoichiometry in water

Figure S2. Nonoptimized initial geometries with β-CD and 5-FU in 1:2 stoichiometry before the four MD runs, lasting for 2 ns, in water starting with two 5-FU drug molecules close to the β-CD primary rim (see panels (a) and (c) in a perpendicular and parallel arrangement near the primary rim, respectively) or initially near the secondary rim (see panels (b) and (d) in a parallel and perpendicular arrangement near the secondary rim, respectively)). Color code in all panels is the same as in Figure S1……………………S4

Video S2: The four MD runs obtained starting from the optimized geometries in Figure S3 are reported in file.avi generated using the Materials Studio program, visualizing the conformations saved every 5 ps during MD runs lasting for 2 ns……………………..S4

Figure S3. Panel (a): the distances between the c.o.m.s of the two 5-FU molecules calculated for the performed MD runs. Panel (b): an instantaneous nonoptimized frame saved during the first MD run at 860 ps (distance calculated: 11.3 Å) in which the distance between -CH of the first 5-FU molecule from the c.o.m. of the second 5-FU drug is equal to 2.698 Å. Panel (c): the instantaneous nonoptimized frame saved during the second MD run at 1685 ps (distance calculated: 8.3 Å) in which the distance between the fluorine atom in the 5-FU molecule from the hydrogen atom in the -CH group of the other drug is equal to 3.169 Å…………………S5

Figure S4. Panels (a), (b), (c) and (d): the mean square displacement (in $Å^2$) related to the two different 5-FUs interacting with the β-CD as a function of the time and the two best linear fit lines that pass through the origin of the Cartesian axes calculated for the two MD runs studied, respectively……….………….………….………….………….………….………….………….………….………….…………S6

Table S1. Data on the slope of the best linear fit of the MSDs calculated for the four MD runs reported in panels (a) and (b) in Figure S4, as well as the diffusion coefficient (*D*) and R^2…………….………….………….………….………….………….………….……..…S6

2.3. β-CD and two 5-FU molecules in a random arrangement in water: from 1:1 to 1:2 inclusion complex formation with different stabilities

Figure S5. Nonoptimized initial geometry before the MD run lasting for 10 ns, in water, with β-CD and the two 5-FUs initially in a random arrangement in a simulation cell. The color code is the same as in Figure S1. ……………………..……………………………...……S7

Video S3. The MD run obtained starting from the optimized geometries in Figure S5 is reported in file.avi generated by using Materials Studio program, visualizing the conformations saved every 5 ps during MD runs lasting for 10 ns………………………………..S7

Figure S6. Panel (a): potential energy and Coulomb contributions; panel (b): van der Waals energy calculated for the MD run performed, lasting for 10 ns, in water, with β-CD and two 5-FU drug molecules initially in a random arrangement in the simulation cell (see nonoptimized initial geometries in Figure S5)…………………………………………………………………………………………S8

2.1. β-CD/5-FU inclusion complexes in a 1:1 host–guest stoichiometry in water

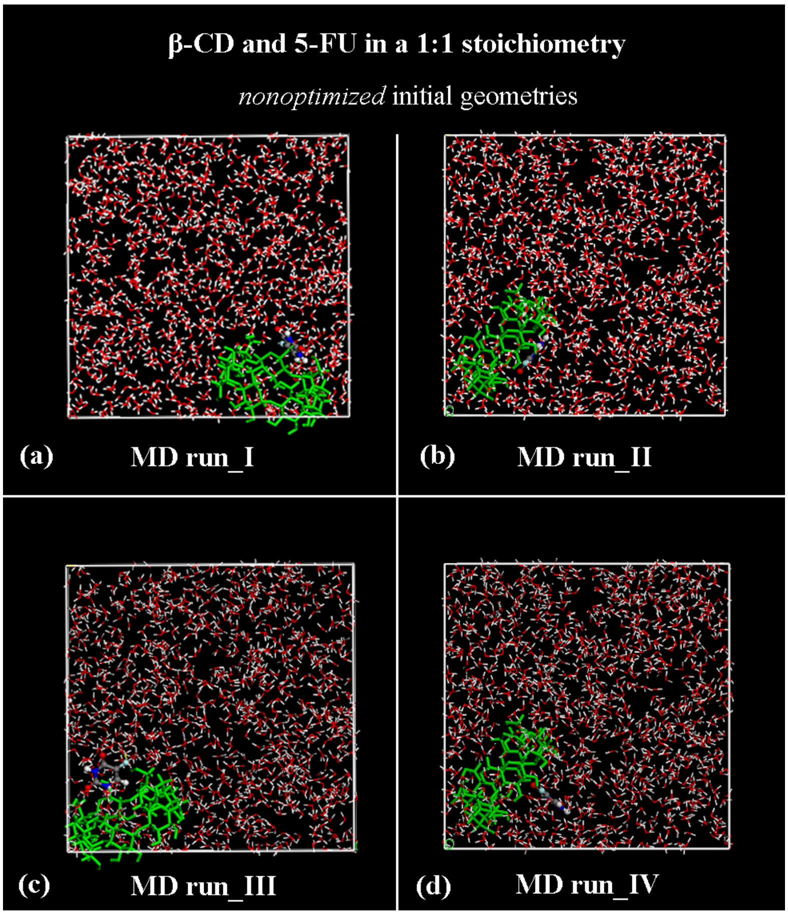

Figure S1. Nonoptimized initial geometries before the four MD runs, lasting for 2 ns, in water, with the 5-FU initially located near the primary rim (see panels (a) and (c)) or near the secondary rim (see panels (b) and (d)). Color code: β-CD carbon atoms are represented by green sticks; carbon atoms of the 5-FU represented by balls and sticks in gray; oxygen in red; nitrogen in blue; fluorine in light blue; hydrogen in white. Water molecules are represented by sticks; oxygen in red; hydrogen in white.

Video S1. The four MD runs obtained starting from the optimized geometries in Figure S1 are reported in file.avi generated using the Materials Studio program, visualizing the conformations saved every 5 ps during MD runs lasting for 2 ns.

1_dyn_5-FU_near Bcd I rim_par_2ns.avi

2_dyn_5-FU_near Bcd II rim_par_2ns.avi

3_dyn_5-FU_near Bcd I rim_perp_2ns.avi

4_dyn_5-FU_near Bcd II rim_perp_2ns.avi

2.1. β-CD/5-FU inclusion complexes in a 1:2 host–guest stoichiometry in water

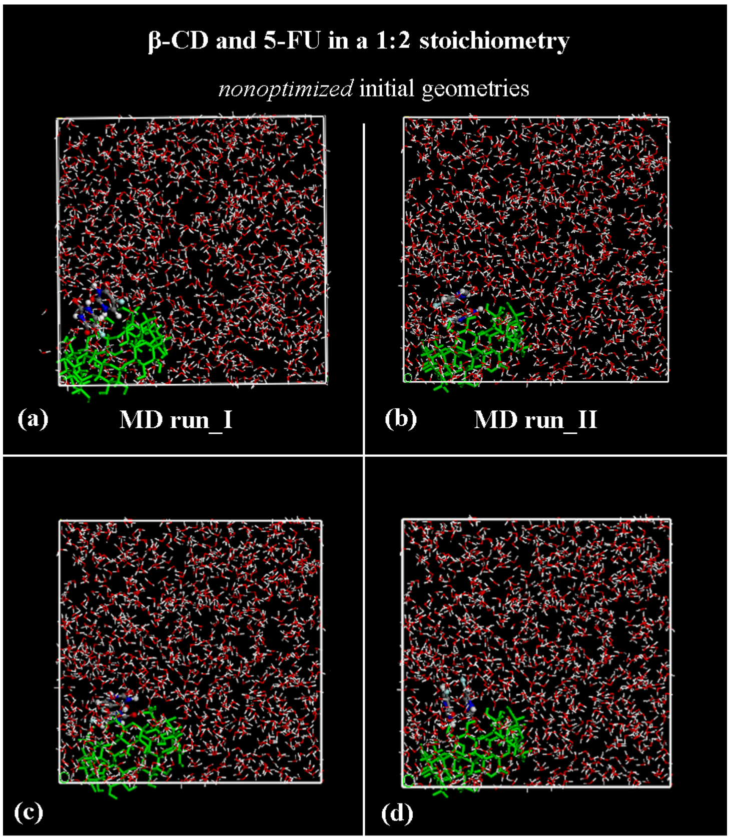

Figure S2. Nonoptimized initial geometries with β-CD and 5-FU in 1:2 stoichiometry before the four MD runs, lasting for 2 ns, in water starting with two 5-FU drug molecules close to the β-CD primary rim (see panels (a) and (c) in a perpendicular and parallel arrangement near the primary rim, respectively) or initially near the secondary rim (see panels (b) and (d) in a parallel and perpendicular arrangement near the secondary rim, respectively). Color code in all panels is the same as in Figure S1.

Video S2: The four MD runs obtained starting from the optimized geometries in Figure S3 are reported in file.avi generated using the Materials Studio program, visualizing the conformations saved every 5 ps during MD runs lasting for 2 ns.

A_dyn_2 5-FU drugs_near Bcd I rim_perp_2ns.avi

B_dyn_2 5-FU drugs_near Bcd II rim_par_2ns.avi

C_dyn_2 5-FU drugs_par Bcd I rim_par_2ns.avi

D_dyn_2 5-FU drugs_par Bcd II rim_perp_2ns.avi

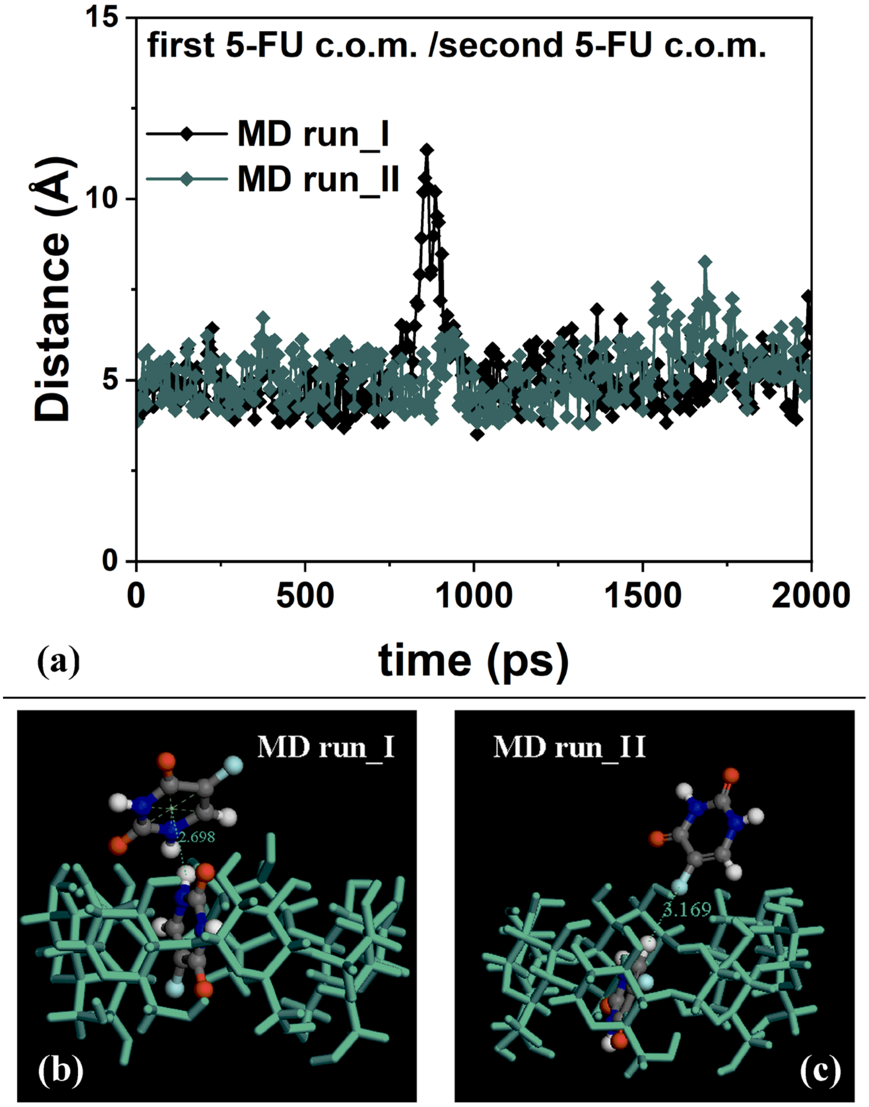

Figure S3. Panel (a): the distances between the c.o.m.s of the two 5-FU molecules calculated for the performed MD runs. Panel (b): an instantaneous nonoptimized frame saved during the first MD run at 860 ps (distance calculated: 11.3 Å) in which the distance between -CH of the first 5-FU molecule and the c.o.m. of the second 5-FU drug is equal to 2.698 Å. Panel (c): the instantaneous nonoptimized frame saved during the second MD run at 1685 ps (distance calculated: 8.3 Å) in which the distance between the fluorine atom in the 5-FU molecule and the hydrogen atom in the -CH group of the other drug is equal to 3.169 Å.

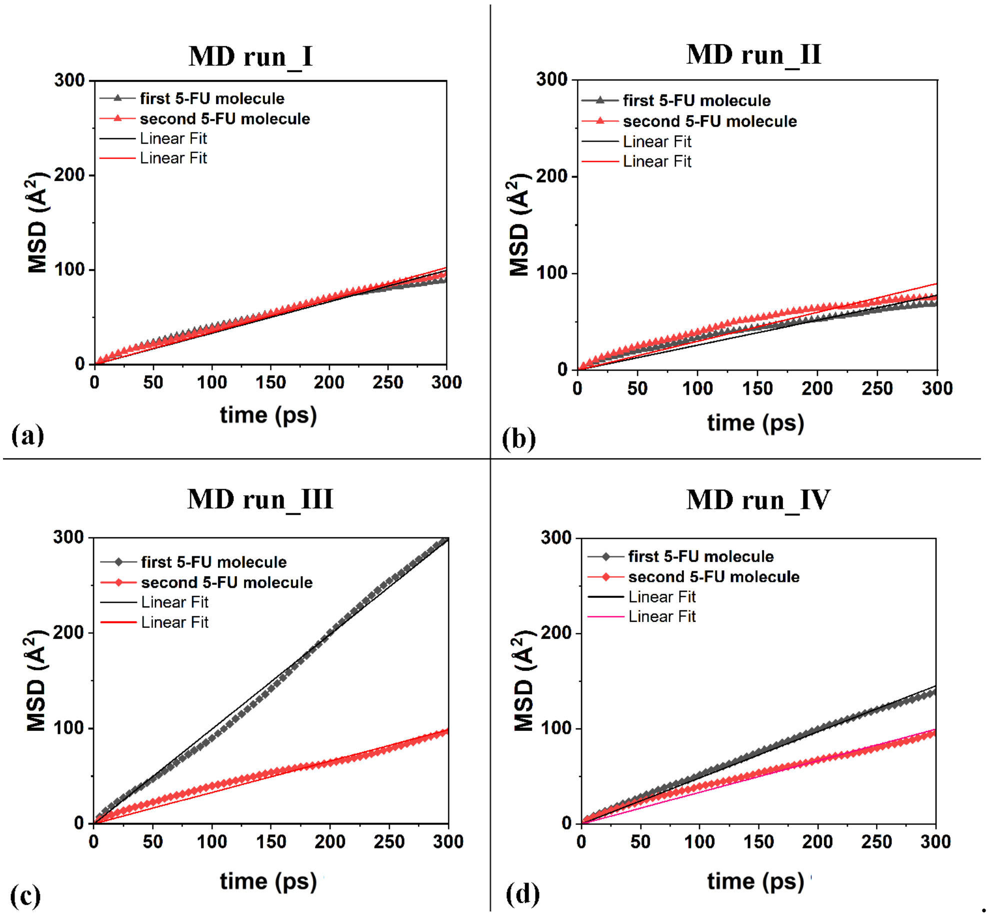

Figure S4. Panels (a), (b), (c) and (d): the mean square displacement (in Å²) related to the two different 5-FUs interacting with the β-CD as a function of the time and the two best linear fit lines that pass through the origin of the Cartesian axes calculated for the two MD runs studied, respectively.

Table S1. Data on the slope of the best linear fit of the MSDs calculated for the four MD runs reported in panels (a) and (b) in Figure S4, as well as the diffusion coefficient (*D*) and R^2.

	**Slope** (Å²/ps)	***D*** (m²/s)	R^2
MD run_I first 5-FU	0.33223 ± 0.00401	5.5372×10^{-10}	0.99118
MD run_I second 5-FU	0.34225 ± 0.00262	5.7042×10^{-10}	0.99643
MD run_II first 5-FU	0.25868 ± 0.00399	4.3113×10^{-10}	0.98567
MD run_II second 5-FU	0.29933 ± 0.00598	4.9888×10^{-10}	0.98803
MD run_III first 5-FU	0.99441±0.00401	1.6573×10^{-9}	0.99901
MD run_III second 5-FU	0.32929 ± 0.00329	5.4882×10^{-10}	0.99395
MD run_IV first 5-FU	0.48372±0.00241	8.0620×10^{-10}	0.99849
MD run_IV second 5-FU	0.33187±0.00326	5.5312×10^{-10}	0.99414

2.3. β-CD and two 5-FU molecules in a random arrangement in water: from 1:1 to 1:2 inclusion complex formation with different stabilities

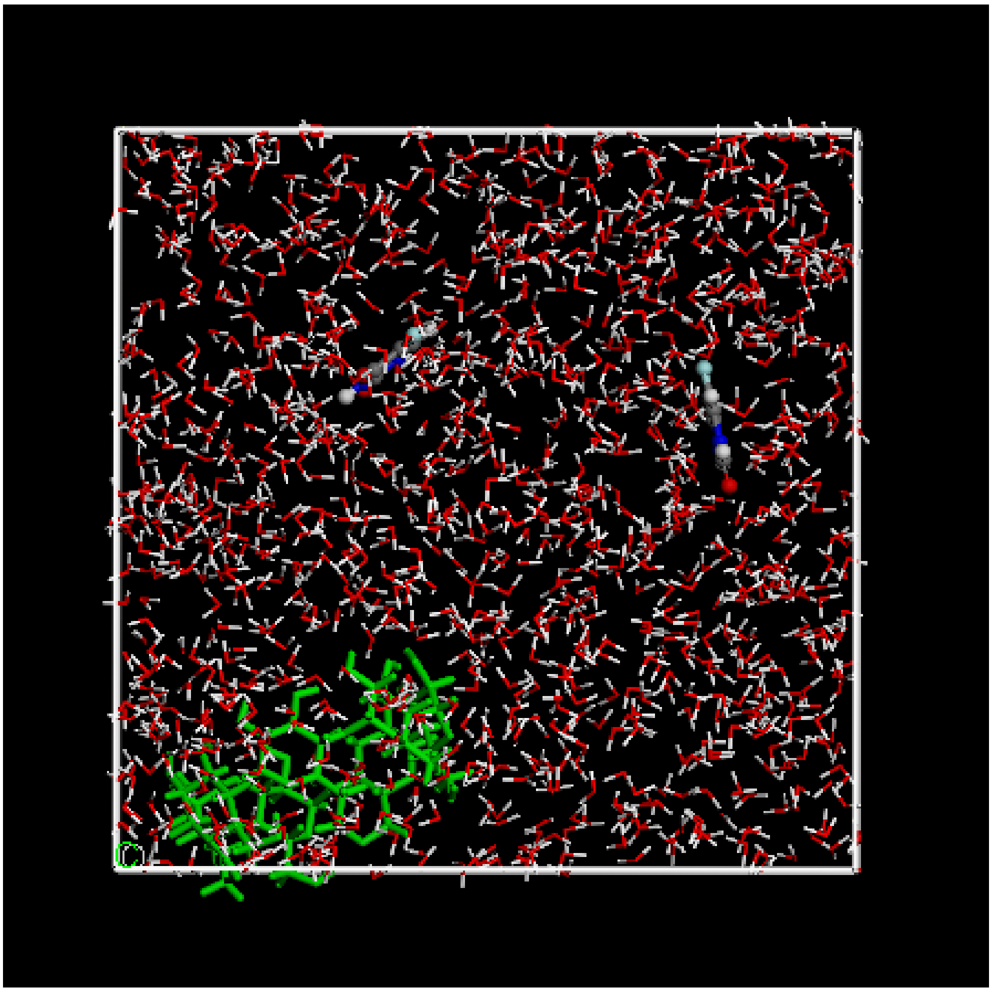

Figure S5. Nonoptimized initial geometry before the MD run lasting for 10 ns, in water, with β-CD and the two 5-FU initially in a random arrangement in a simulation cell. The color code is the same as in Figure S1.

Video S3. The MD run obtained starting from the optimized geometries in Figure S5 is reported in file.avi generated using the Materials Studio program, visualizing the conformations saved every 5 ps during MD runs lasting for 10 ns.

Figure&avi\avi\RANDOM_dyn_Bcd_2FU_rand_aq_10ns.avi

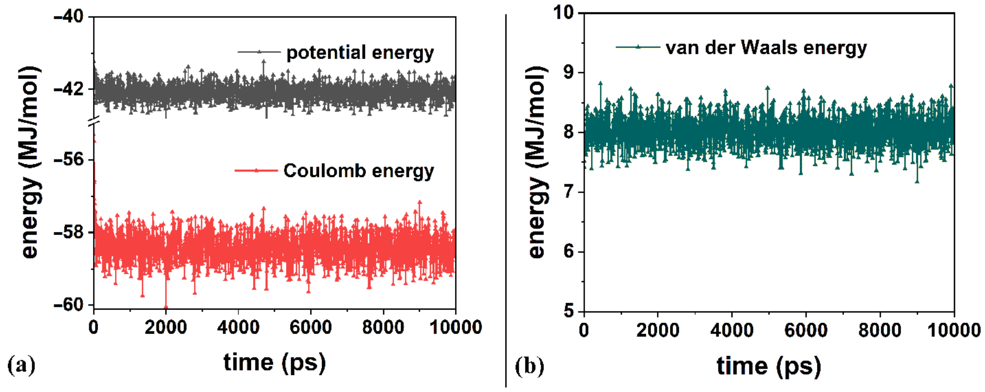

Figure S6. Panel (a): potential energy and Coulomb contributions; panel (b): van der Waals energy calculated for the MD run performed, lasting for 10 ns, in water, with β-CD and two 5-FU drug molecules initially in a random arrangement in the simulation cell (see nonoptimized initial geometries in Figure S5).
